# Multivariate analysis of independent determinants of ADL/IADL and quality of life in the elderly

**DOI:** 10.1186/s12877-022-03621-3

**Published:** 2022-11-23

**Authors:** Sebastian Beltz, Simone Gloystein, Thomas Litschko, Sonja Laag, Neeltje van den Berg

**Affiliations:** 1 Institute for Community Medicine, Section Epidemiology of Health Care and Community Health, University Medicine Greifswald, Greifswald, Germany; 2 Department for Product Strategy/Development, BARMER Health Insurance, Wuppertal, Germany

**Keywords:** Elderly, Disability, ADL, IADL, Quality of life, WHOQOL-BREF, WHOQOL-OLD, Physical health, Social participation, Multivariate analysis

## Abstract

**Background:**

This study evaluated the determinants of disability and quality of life in elderly people who participated at the multi-centred RubiN project (Regional ununterbrochen betreut im Netz) in Germany.

**Methods:**

Baseline data of the subjects aged 70 years and older of the RubiN project were used and only subjects with complete data sets were considered for the ensuing analysis (complete case analysis (CCA)).

Disability was examined using the concepts of ADL (activities of daily living) and IADL (instrumental activities of daily living). Subjects exhibiting one or more deficiencies in ADL respectively IADL were considered as ADL respectively IADL disabled. Quality of life was assessed using the WHOQOL-BREF and the WHOQOL-OLD. Applying multivariate analysis, sociodemographic factors, psychosocial characteristics as well as the functional, nutritional and cognitive status were explored as potential determinants of disability and quality of life in the elderly.

**Results:**

One thousand three hundred seventy-five subjects from the RubiN project exhibited data completeness regarding baseline data. ADL and IADL disability were both associated with the respective other construct of disability, sex, a reduced cognitive and functional status as well as domains of the WHOQOL-BREF. Furthermore, ADL disability was related to social participation, while IADL disability was linked to age, education and social support. Sex, ADL and IADL disability, income, social support and social participation as well as the functional status were predictors of the domain ‘Physical Health’ (WHOQOL-BREF). The facet ‘Social Participation’ (WHOQOL-OLD) was affected by both ADL and IADL disability, income, social participation, the nutritional and also the functional status.

**Conclusions:**

Several potential determinants of disability and quality of life were identified and confirmed in this study. Attention should be drawn to prevention schemes as many of these determinants appear to be at least partly modifiable.

**Supplementary Information:**

The online version contains supplementary material available at 10.1186/s12877-022-03621-3.

## Introduction

As life expectancy of the global population is increasing, the term Aging is of growing interest in scientific literature [1, 2]. In Germany, the population of people aged 70 years and older rose from 8.1 million in 1990 to 13.4 million in 2020, and is predicted to reach 18.0 million in 2050 if assuming a moderate development of life expectancy and a low net migration. This constitutes a percentage share of people aged 70 years and older of 10% in 1990, 16% in 2020 and 23% in 2050 respectively [3]. Research has extensively discussed possible consequences of an aging population which led to the two contrasting concepts of compression vs. expansion of morbidity. The former comprises the ‘ideal of a long life with a short period of terminal decline’ [4]. Whereas the latter hypothesises that an advanced medical technology will primarily reduce fatal diseases in contrast to nonfatal diseases leading to a promotion of morbidity while diminishing mortality [5]. In fact, literature offers evidence for both theories [6]. This lack of an unequivocal statement in favour of one of the both concepts is probably due to the nature of aging: It is a complex, dynamic and heterogeneous process [6–8]. While some individuals manage to maintain their functional status which is considered as ‘successful aging’ or ‘healthy aging’, others experience what is known as ‘usual aging’ [8–10]. ‘Usual aging’ connotes an enhanced risk of multimorbidity and a subsequent increase in disability as well as a reduction in functional status and quality of life (QOL), leading to augmented health care costs [11, 12]. According to the World Health Organization’s ICF Model (International Classification of Functioning, Disability and Health), functioning and disability are the outcome of an interplay between a person’s health status and individual influencing variables such as the environment and personal factors. Thus, disability occurs if the interaction of these factors negatively results in straitened activities and/or participation [13]. Hence the development of disability is deemed to be an individual, complex, multifactorial process usually occurring over a course of several years [14–18]. Moreover disability depends on the respective sociocultural context, and should be assessed according to it [17, 19]. The scope of disability depends on the abilities affected by impairment and the level of this given impairment [17]. Scientific research commonly assesses a person’s disability by using the concepts of activities of daily living (ADL) and instrumental activities of daily living (IADL) [20, 21]. ADL comprises activities indispensable for an independent self-care (feeding, transfer, grooming, toilet use, bathing, walking, stair climbing, dressing and undressing as well as fecal and urinary incontinence) [22, 23]. In contrast IADL refers to abilities needed for an autonomous interaction with the environment (using the telephone, shopping, preparing meals, housekeeping, doing the laundry, moving within the community, taking prescribed medications as well as managing finances) [24, 25]. Considering people aged 70 years and older, studies reported a prevalence of ADL-disability ranging from 13% to approximately 40% [26–28] and a prevalence of IADL-Disability ranging from 27% to roughly 40% [26, 28]. It is noteworthy that levels of disability significantly vary between different parts of the world, with the lowest levels observed in Northern and Western Europe [29]. The sociocultural context may also decide which ADL/IADL items are most likely impaired [30]. While ADL primarily rely on physical and health-related factors, IADL are more strongly related to psychosocial, cognitive and economic resources [31–33]. Moreover, a decline in IADL usually precedes limitations in ADL, and may therefore serve as a predictor for future ADL-decline [22, 34]. Conversely, independence in ADL acts as a precondition for autonomy in terms of IADL [31, 32]. Although a limitation in ADL and/or IADL may be partly reversible [35, 36], disability is known to have adverse effects. These include a loss of autonomy and dependence on either formal or informal care [37, 38], institutionalisation [39, 40], an increase in mortality rates [41, 42] and health care costs [38] as well as a decline in QOL [43–45]. QOL is defined by the WHO as an ‘individuals’ perception of their position in life in the context of the culture and value systems in which they live and in relation to their goals, expectations, standards and concerns’ [46]. It is recognized as a multifactorial, highly subjective construct which is again affected by a person’s individual socio-cultural context [47–51]. In recent years, particularly health-related quality of life (HRQOL) has become a matter of interest [51, 52], and is commonly used as a clinical endpoint, when evaluating the outcomes of medical procedures [47, 53].

Due to the significance of disability and QOL research intensively investigated possible determinants regarding functional status and QOL, in order to promote prevention and rehabilitation schemes [54–58]. Depending on the socio-cultural context frequently observed determinants of disability and QOL are: social demographics, housing situation, socioeconomical status, psychosocial characteristics (social participation, social support), health behaviour, relevant comorbidities, self-rated health as well as the reciprocal interaction of disability and QOL [20, 54, 58–66]. Regarding the aforementioned determinants, older age represents a risk factor regarding disability [67, 68], while its influence on QOL remains controversial [47, 49, 69]. Female sex is found to negatively affect disability and QOL [44, 70–72]. However, it was suggested that both older age and female sex only exert a significant influence on disability and QOL, when acting as mediator variables for other unfavourable risk factors mentioned below. After adjusting for other confounding variables, age and sex partly became insignificant [50, 73–75]. In addition, the following variables are predominantly perceived as risk factors concerning both disability and QOL: being single/unmarried and living without a partner [71, 76–78], facing constraints regarding socioeconomical status [27, 79–81], as well as social participation and support [20, 82–84], malnutrition and under- or overweight [70, 85, 86], limited physical activity [68, 87, 88], comorbidities [68, 87, 89, 90] and a low self-rated health status [49, 91, 92]. Moreover, disability serves as a risk factor for QOL, while a reduced QOL also poses a threat to independent functioning [31, 45, 68, 93].

In this paper we present novel insight about the determinants of disability and QOL in a real German ambulant healthcare setting. There are several reasons for this approach. First, to our best knowledge those determinants are usually assessed in mere cross-sectional or cohort studies, but have not been investigated in an actual care setting yet [94–98]. Second, determinants vary between different sociocultural contexts and distinct health policy systems [19, 29, 41]. This holds true even when comparing countries within Western and Northern Europe [99]. When aiming to improve the health care situation of German geriatric patients, it is therefore important to ascertain the actual determinants in a German setting. Third, a complete geriatric assessment is time-consuming and demanding for elderly patients. This has been shown to delay the start of intervention and treatment [100]. The knowledge of specific determinants will help detecting geriatric patients with needs and individuals at risk of developing disability more rapidly [101]. Considering the dynamic nature of disability, an earlier detection of affected patients will promote prevention, intervention and restoration [55]. Forth, as there is still controversy about particular determinants of disability and QOL, this study might provide further insight [102].

## Methods

### Study population

Data used in this study were collected in the context of the RubiN project. This is a prospective controlled intervention study in an ambulant clinical setting (i.e. geriatric patients of general practices) which was set up to assess the impact of case management for elderly patients [103]. The Ethics Committee of the University of Greifswald approved the study (Application number: BB188/18). In total eight certified practice networks participated in the RubiN project. Five networks served as intervention group, while the other three made up the control group. All patients of the eight practice networks who were aged 70 and over completed the Angelina questionnaire, a screening tool comprising the most important geriatric issues (housing and assistance needs, medication, mobility, senses, recent medical history, cognition, mood) [104]. This questionnaire is available in German only. All of these patients displaying at least two different areas of geriatric need according to the Angelina questionnaire were eligible for participation according to the study’s inclusion criteria. Exclusion criteria were: receiving inpatient or specialised palliative care, having a terminal illness or a bipolar disorder as well as other severe psychiatric medical conditions. Considering the study’s inclusion and exclusion criteria and withdrawal of informed consent, baseline assessment data from 4116 subjects was available. A detailed description of the original study’s sample size calculation can be found elsewhere [103]. After replacing missing items in the WHOQOL questionnaires according to the respective manual [105, 106], 1375 subjects with complete baseline data sets were considered for the ensuing analysis (CCA). There was no imputation of data and no controlled missings were permitted.

### Outcome

The outcome variables in this study were disability and QOL.

Disability was examined applying the Barthel index (BI) for ADL according to the Hamburg Classification Manual [107] and the Lawton and Brody scale for IADL [24, 25]. The BI is a valid geriatric assessment measure, and the Hamburg Classification Manual is considered to be the standard operationalisation of the BI in German geriatrics [108, 109]. The BI contains ten items with maximum item scores ranging from five to fifteen depending on the specific item. Full dependence concerning an item is equated with a score of zero. If a person exhibits full dependence in one or more items (i.e., attainment of one or more scores of zero), the person is identified as ADL disabled [110, 111]. Hence, study participants were dichotomised as ADL disabled or ADL independent. The Lawton and Brody scale for IADL is a valid and reliable assessment tool including eight items [112]. An item score of one indicates mastery of the respective task, while an item score of zero displays dependency regarding the particular item. Study participants achieving the maximum total score of 8 were considered as IADL independent, while subjects with one or more IADL dependencies were identified as IADL disabled [7, 71].

QOL was assessed using the WHOQOL-BREF and the WHOQOL-OLD. The WHOQOL-BREF is a valid and reliable short version of the WHOQOL-100 [113–115]. It contains the four domains ‘Physical Health, ‘Psychological’, ‘Social Relationships’ and ‘Environment’ which are evaluated with a total of 24 items. In addition, the WHOQOL-BREF includes two general items assessing the global QOL as well as the contentment with the current self-perceived health status (i.e., the ‘Global Score’). The WHOQOL-OLD is commonly used as add-on instrument in a geriatric setting in order to cover specificities regarding the QOL of elderly people [51, 116]. It is composed of six facets, each comprising four items. The six facets are: ‘Sensory Abilities’, ‘Autonomy’, ‘Past, Present and Future Activities’, ‘Social Participation’, ‘Death and Dying’, ‘Intimacy’. The mean of those six facets forms the ‘Total Score’ of the WHOQOL-OLD. For both the WHOQOL-BREF and the WHOQOL-OLD, each item was rated on a 5-point Likert scale. Mean scores of each domain/facet were then transformed into a scale with values ranging from 0 to 100 with higher scores indicating greater levels of QOL [62, 117].

### Determinants

The independent variables were chosen based on a preceding literature review and availability in the RubiN project. Regarding sociodemographic determinants age and sex were recorded. Age was used as a continuous variable [118]. The living situation was assessed, and divided into three categories: living alone, living with a spouse/partner, living with others than a spouse/partner in a private house [40]. Place of residence was dichotomised into living in an urban (≥ 20,000 inhabitants) or in a rural area (< 20,000 inhabitants) [110]. Education was recorded, and partitioned into three categories: high (higher education entrance qualification and equivalent), intermediate (attending school for 10 years and equivalent) and low level (attending school for less than 10 years) [119]. Income was examined as well. As subjectivity is deemed to be more important than objective measures in financial matters [49, 69, 120], study participants were asked to subjectively rate their current income as ‘appropriate’, ‘scarce’ or ‘not enough for a living’. The latter two categories were pooled leaving two categories: Those with and those without financial difficulties [110]. Social support was divided into two categories: enjoying support (family, caregivers, others) or lacking support [47]. Social participation was assessed by two questions (meeting with friends, leisure activities). If subjects declared a shortage in any of those areas, they were viewed as lacking social participation [87, 102]. Nutritional status was examined using the Mini Nutritional Assessment (MNA). It contains 18 items, and encompasses four domains: anthropometric, global, dietary and self-assessment. The maximum total score is 30. Scores between 24 and 30 express a normal status, while scores between 17 and 23.5 indicate a risk of malnutrition. Scores less than 17 signal malnutrition [121]. The cognitive status was examined using the DemTect, a valid, reliable and highly-sensitive screening tool for (mild) cognitive impairment and different types of dementia. The DemTect contains five tasks: transcoding numbers, a semantic verbal fluency task, a numerical sequence in reverse order as well as a word list and a delayed recall of the word list. A maximum score of 18 points can be attained. 13 to 18 points imply an age-appropriate status, 9 to 12 points display a mild cognitive impairment and less than 9 points indicate possible dementia [122, 123]. Finally, functional status was tested using the Timed Up-and-Go Test (TUG). It has been proven to be a valid and reliable screening tool for lacking balancing skills and consequently for increased risk of falls. The TUG quantifies the time needed for a person to rise from a chair, walk three meters, return and to sit back down [124, 125]. As a whole variety of cut-off values for the TUG had been used in previous studies [126], this study adopted the age-specific reference values proposed in a meta-analysis: 10.2 seconds (70–79 years old) and 12.7 seconds (≥ 80 years old) [127]. Using these cut-off values, subjects were classified as better or worse than the age-specific cut-off value.

### Statistical analysis

The statistical analysis was carried out using JMP Pro 15 (SAS Institute, Cary, North Carolina, USA).

First, we examined the study participants’ descriptive characteristics regarding the frequencies, means and standard deviations of the dependent and independent variables. Descriptive analysis was performed for the complete study sample as well as for those subjects included and excluded for the subsequent analysis. Second, bivariate analysis was performed. Independent variables of ADL and IADL were tested using Chi^2^-tests based on logistic regression for continuous variables and contingency analysis for categorial variables. Independent variables of QOL were assessed using bivariate correlation for continuous variables and two-sided pooled t-tests respectively ANOVAs for categorial variables. Third, multiple regression models were created in order to identify and confirm independent variables significantly influencing ADL, IADL and QOL (WHOQOL-BREF and -OLD) and to examine the relationship between the outcome variables. The dichotomous dependent variables ADL and IADL were examined using nominal logistic regression, whereas the continuous dependent variables of QOL (domains, facets and global/total scores) were assessed using mean least squares regression. Regression models were tested towards their Lack of Fit and significance, the explained variance R^2^ was computed. Independent variables within each regression model were analysed using Effect Likelihood Ratio Tests, and significant Parameter Estimates were identified. Odds ratios were calculated for the categorial dependent variables ADL and IADL. For all statistical tests a *p*-value < 0.05 was considered statistically significant.

## Results

Table 1 displays the descriptive statistics of the whole study sample (*N* = 4116), thereof *N* = 1375 persons could be included in the analysis. *N* = 2741 persons had to be excluded due to missing data. Executed bivariate analysis indicates that there are several significant differences regarding the independent variables between the two latter groups. For example, excluded subjects were significantly older, exhibited larger proportions of ADL respectively IADL disabled persons, rated their quality of life significantly lower in most of the domains of the WHOQOL-BREF and the WHOQOL-OLD and performed worse on the Timed Up-and-Go test.

**Table 1:** Descriptive Analysis for the total sample, included and excluded participants

Variable	Total sample (*N* = 4116)		Included (***N*** = 1375)		Excluded (***N*** = 2741)		*p* value
**Age (years)**	*M*	*SD*	*M*	*SD*	*M*	*SD*	
	81.6	5.7	80.7	5.6	82.1	5.8	< 0.0001* ^1^
**Women (%)**	64		64		64		0.8950 ^3^
**ADL disabled (%)**	36		25		42		< 0.0001* ^3^
**IADL disabled (%)**	57		46		63		< 0.0001* ^3^
**WHOQOL-BREF**	*M*	*SD*	*M*	*SD*	*M*	*SD*	
**Physical Health**	55.4	19.7	57.9	18.8	51.9	20.3	< 0.0001* ^2^
**Psychological**	63.8	16.2	66.0	14.8	60.7	17.4	< 0.0001* ^2^
**Social Relationships**	67.4	16.6	68.1	16.1	66.5	17.2	0.0190* ^1^
**Environment**	69.5	14.3	70.9	13.7	67.4	14.9	< 0.0001* ^2^
**WHOQOL-BREF: Global Score**	56.0	20.2	58.3	19.2	52.9	21.0	< 0.0001* ^2^
**WHOQOL-OLD**							
**Sensory Abilities**	65.8	22.0	68.3	20.9	62.3	23.1	< 0.0001* ^2^
**Autonomy**	60.5	19.5	62.3	18.8	58.0	20.2	< 0.0001* ^2^
**Past, Present and Future Activities**	61.7	15.7	63.0	14.8	60.0	16.8	< 0.0001* ^2^
**Social Participation**	57.3	20.5	59.9	18.9	53.7	22.0	< 0.0001* ^2^
**Death and Dying**	69.6	22.5	69.4	22.2	69.9	22.7	0.5806 ^1^
**Intimacy**	66.8	19.5	67.5	18.7	65.7	20.4	0.0333* ^2^
**WHOQOL-OLD Total Score**	63.6	13.4	65.1	12.5	61.6	14.3	< 0.0001* ^2^
**Living Situation (%)**							< 0.0001* ^3^
**Living alone**	39		40		39		
**Living with a partner**	50		53		49		
**Living with others**	10		7		12		
**Rural (%)**	41		38		42		0.0459* ^3^
**Education (%)**							0.1316 ^3^
**Low**	73		71		74		
**Intermediate**	17		18		17		
**High**	10		10		9		
**Income (subjective) (%)**							0.0015* ^3^
**Appropriate**	80		83		79		
**Social Support (available) (%)**	86		84		87		0.0068* ^3^
**Social Participation (no shortage) (%)**	71		67		73		< 0.0001* ^3^
**MNA (%)**							< 0.0001* ^3^
**< 17**	3		1		4		
**17–23.5**	15		11		17		
**≥ 24**	82		88		78		
**DemTect (%)**							0.1259 ^3^
**< 9**	14		13		14		
**9–12**	30		29		30		
**13–18**	57		59		55		
**Timed Up-and-Go (%)**							
**Better than age-specific** **cut-off value**	39		46		35		< 0.0001* ^3^

Data: means and standard deviations for continuous variables – *M* mean, *SD* standard deviation; percentages for categorial variables, rounded down/up if applicable – if a variable consists of only two categories just one is displayed, missing values were excluded prior to the calculation of each variable; *p* values for differences between included and excluded study participants; ^1^ two-sided pooled t-test, ^2^ Welch’s t-test – choice of tests based on test results for homoscedasticity (Brown-Forsythe), ^3^ Chi^2^-tests, *p* <  0.05

The 1375 study participants included in this analysis had a mean age of 80.7 ± 5.6 years with a female percentage share of 64% (women: *N* = 877, men: 498). 24.8% of the included study participants were ADL disabled, while 45.8% were IADL disabled. Sex did not exert a significant influence on ADL disability (women: 26.45%; men: 21.89%), while significantly more men than women were IADL disabled (women: 42.42%; men: 51.81%).

### ADL and IADL

After multivariate analysis (data partly not shown – an additional file shows this analysis in more detail (see Additional file 3), see Table 2), eight variables remained as significant determinants of ADL disability: female sex, IADL disability, higher scores regarding ‘Environment’ and lower scores concerning ‘Physical Health’ and the ‘Global Score’ (WHOQOL-BREF), sufficient social participation as well as a reduction in cognitive and functional status. With regard to the Odds Ratios, a prevalent IADL disability was the dominant risk factor (OR: 7.77, see Table 3).

**Table 2:** Multivariate analysis - parameter estimates for ADL disability and IADL disability

Variable	ADL disability		IADL disability	
	β (95% CI)	*p* value	β (95% CI)	*p* value
**Intercept**	−0.56 (− 3.21–2.09)	0.6791 ^1^	−1.13 (− 3.87–1.61)	0.4200 ^1^
**Age (continuous)**	–	–	0.06 (0.03–0.09)	0.0002* ^1^
**Women**	0.27 (0.08–0.46)	0.0053* ^1^	− 0.33 (− 0.50 - -0.16)	0.0002* ^1^
**ADL disabled**	–	–	1.02 (0.81–1.24)	< 0.0001* ^1^
**IADL disabled**	1.02 (0.81–1.24)	< 0.0001* ^1^	–	–
**WHOQOL-BREF**				
**Physical Health**	−0.02 (− 0.03 - -0.01)	0.0027* ^1^	− 0.03 (− 0.04 - -0.01)	0.0002* ^1^
**Psychological**	–	–	–	–
**Social Relationships**	–	–	0.01 (0.003–0.03)	0.0140* ^1^
**Environment**	0.02 (0.002–0.04)	0.0325* ^1^	−0.03 (− 0.05 - − 0.01)	0.0017* ^1^
**Global Score**	-0.01 (− 0.03 - -0.003)	0.0148* ^1^	–	–
**WHOQOL-OLD**				
**Sensory Abilities**	–	–	–	–
**Autonomy**	–	–	–	–
**Past, Present and Future Activities**	–	–	–	–
**Social Participation**	–	–	–	–
**Death and Dying**	–	–	–	–
**Intimacy**	–	–	–	–
**Total Score**	–	–	–	–
**Living Situation**	–	–	–	–
**Rural-Urban**	–	–	–	–
**Education**	–	–		
**Intermediate**			−0.29 (−0.57 - -0.01)	0.0437* ^1^
**Income (subjective)**				
**Appropriate**	–	–	–	–
**Social Support (available)**	–	–	0.57 (0.34–0.80)	< 0.0001* ^1^
**Social Participation** **(no shortage)**	0.18 (0.01–0.36)	0.0430* ^1^	–	–
**MNA** **< 17** **≥ 24**	–	–	–	–
**DemTect** **< 9**	0.31 (0.03–0.59)	0.0314* ^1^	0.43 (0.10–0.76)	0.0105* ^1^
**Timed Up-and-Go**				
**Better than age-specific** **cut-off value**	−0.33 (− 0.52 - -0.13)	0.0009* ^1^	− 0.27 (− 0.44 - -0.11)	0.0007* ^1^

^1^ Chi^2^-tests based on nominal-logistic regression for log odds of ADL disabled/ADL independent, ^2^ two-sided t-tests based on mean least squares regression, *p* <  0.05. The R^2^ of the regression model was 0.34 for ADL and 0.39 for IADL

**Table 3:** Multivariate analysis - odds ratio for ADL disability and IADL disability

Variable	ADL disability		IADL disability	
	OR (95% CI)	*p* value	OR (95% CI)	*p* value
**Age** **(per additional year of age)**	–	–	1.06 (1.03–1.09)	0.0002* ^1^
**Women**	1.72 (1.17–2.52)	0.0053* ^1^	0.52 (0.37–0.73)	0.0002* ^1^
**ADL disabled**	–	–	7.76 (5.06–11.90)	< 0.0001* ^1^
**IADL disabled**	7.77 (5.09–11.86)	< 0.0001* ^1^	–	–
**WHOQOL-BREF**				
**Physical Health**	0.98 (0.97–0.99)	0.0027* ^1^	0.97 (0.96–0.99)	0.0002* ^1^
**Psychological**	–	–	–	–
**Social Relationships**	–	–	1.01 (1.00–1.03)	0.0140* ^1^
**Environment**	1.02 (1.00–1.04)	0.0325* ^1^	0.97 (0.96–0.99)	0.0017* ^1^
**Global Score**	0.99 (0.97–1.00)	0.0148* ^1^	–	–
**WHOQOL-OLD**				
**Sensory Abilities**	–	–	–	–
**Autonomy**	–	–	–	–
**Past, Present and Future Activities**	–	–	–	–
**Social Participation**	–	–	–	–
**Death and Dying**	–	–	–	–
**Intimacy**	–	–	–	–
**Total Score**	–	–	–	–
**Living situation**	–	–	–	–
**Rural**	–	–	–	–
**Education** **Low vs. intermediate**				
	–	–	1.52 (1.02–2.26)	0.0392* ^1^
**Income (subjective)** **Appropriate**				
	–	–	–	–
**Social Support (available)**	–	–	3.15 (1.99–4.97)	< 0.0001* ^1^
**Social Participation (no shortage)**	1.44 (1.01–2.04)	0.0430* ^1^	–	–
**MNA**				
**≥ 17–23.5 vs. ≥ 24**	1.70 (1.09–2.65)	0.0190* ^1^	2.03 (1.20–3.42)	0.0079* ^1^
**DemTect**				
**< 9 vs. 13–18**	2.06 (1.29–3.28)	0.0025* ^1^	2.36 (1.41–3.96)	0.0011* ^1^
**9–12 vs. 13–18**	1.66 (1.15–2.40)	0.0064* ^1^	1.54 (1.09–2.16)	0.0134* ^1^
**Timed Up-and-Go**				
**Better than age-specific** **cut-off value**	0.52 (0.35–0.77)	0.0009* ^1^	0.57 (0.41–0.79)	0.0007* ^1^

^1^ Chi^2^-tests based on nominal-logistic regression, *p* <  0.05; *OR *Odds Ratio, *CI *confidence interval, *OR *Odds Ratio for the odds of being ADL disabled respectively IADL disabled

Significant determinants of IADL disability were an older age, male sex, ADL disability, a lower rating regarding ‘Physical Health’ and ‘Environment’ as well as a higher rating in terms of ‘Social Relationships’ (WHOQOL-BREF), having social support and a reduced cognitive and functional status. Attainment of an intermediate education level served as a protective factor regarding IADL disability, while a higher education level did not exert a significant influence. Analogous to ADL disability, the most important determinant of IADL disability was a given ADL disability (OR: 7.76, see Table 3).

### WHOQOL-BREF and WHOQOL-OLD

Multivariate analysis displayed the different significant factors of quality of life (data not shown, an additional file shows this analysis in more detail (see Additional file 3)). Significant determinants for a decrease in the domain ‘Physical Health’ were female sex, a prevalent ADL respectively IADL disability, an insufficient income, having social support and a lack of social participation as well as a reduced functional status (see Table 4). ADL respectively IADL disability also yielded a significant negative influence on the domain ‘Social Participation’ alongside scarcity of an adequate income and social participation as well as a reduced nutritional and functional status (see Table 4).

**Table 4:** Multivariate analysis – parameter estimates for the domain/facet of WHOQOL-BREF and WHOQOL-OLD with the highest R^2^

Variable	Physical Health (WHOQOL-BREF)		Social Participation (WHOQOL-OLD)	
	β (95% CI)	*p* value	β (95% CI)	*p* value
**Intercept**	65.84 (52.95–78.74)	< 0.0001* ^1^	59.27 (47.75–72.93)	< 0.0001* ^1^
**Age (continuous)**	–	–	–	–
**Women**	−1.80 (−2.70 - -0.88)	0.0001* ^1^	–	–
**ADL disabled**	−3.29 (−4.39 - -2.20)	< 0.0001* ^1^	− 3.55 (− 4.70 - -2.40)	< 0.0001* ^1^
**IADL disabled**	− 4.09 (− 5.09 - -3.09)	< 0.0001* ^1^	− 3.97 (− 5.02 - -2.92)	< 0.0001* ^1^
**Living Situation**	–	–	–	–
**Rural-Urban**	–	–	–	–
**Education** **Intermediate**	–	–	–	–
**Income (subjective)**				
**Appropriate**	1.51 (0.42–2.59)	0.0065* ^1^	1.49 (0.36–2.63)	0.0101* ^1^
**Social Support (available)**	−1.24 (−2.36 - -0.12)	0.0296* ^1^	–	–
**Social Participation** **(no shortage)**	3.73 (2.85–4.60)	< 0.0001* ^1^	4.45 (3.53–5.36)	< 0.0001* ^1^
**MNA**	–	–		
**< 17**			−9.72 (−15.52 - -3.92)	0.0010* ^1^
**≥ 24**			6.62 (3.52–9.72)	< 0.0001* ^1^
**DemTect** **< 9**	–	–	–	–
**Timed Up-and-Go** **Better than age-specific** **cut-off value**	5.44 (4.54–6.34)	< 0.0001* ^1^	3.53 (2.59–4.47)	< 0.0001* ^1^

^1^ Two-sided t-tests based on mean least squares regression, *p* < 0.05. The R^2^ of the regression model was 0.38 for ‘Physical Health’ (WHOQOL-BREF) and 0.32 for ‘Social Participation’ (WHOQOL-OLD)

## Discussion

The significant differences between the 2741 excluded and the 1375 included study participants suggest the presence of a healthy participation effect [111, 128]. The consequence might be an underestimation of disability, an overestimation of QOL and an alteration of the influential factors of both disability and QOL [80, 99]. In turn this could be the reason for variables like ‘Living Situation’ and ‘Rural-Urban’ apparently being insignificant in multivariate analysis despite their relative importance in literature [97].

The reported prevalence of ADL (24.8%) and IADL disability (45.8%, see Table 1) among the elderly implies higher disability rates than found in comparable settings in Ireland (ADL: 13%, IADL: 11%) and Israel (ADL: 6%, IADL: 20%) [68, 129]. Differences in disability levels may stem from specific risk factors in a community or country, divergent health care policies as well as sociocultural factors including different gender roles and distinct confession of disability [19, 30, 96, 99]. As the percentage share of IADL disability exceeds ADL disability in this sample, this may support previous findings of IADL disability preceding ADL disability due to the more complex nature of IADL [22, 31, 34].

The QOL in this study population in terms of the WHOQOL-BREF was considerably higher than in a similar Polish sample (‘Physical Health’: 57.9 vs. 50.9, ‘Psychological’: 66.0 vs. 59.2, ‘Social Relationships’: 68.1 vs. 54.8, ‘Environment’: 70.9 vs. 58.5) [66]. Unfortunately, the majority of previous studies using the WHOQOL questionnaires included subjects aged 60 years and older, hampering a comparison with this study’s sample [47, 62, 130, 131]. The same applies to the German reference data from the WHOQOL-OLD manual [117]. Nevertheless, the independent variable age seems to have a wide influence on QOL with higher age usually being related to a diminished QOL [118, 132].

Multivariate analysis revealed that both ADL and IADL disability were significantly associated with the respective other type of disability: IADL disability was the strongest predictor for ADL disability (OR: 7.77) and vice versa (OR: 7.76). A sizeable intercorrelation between both concepts is in line with previous findings and emphasizes that independence in ADL/IADL serves as a prerequisite for the respective other one [23, 31]. Nevertheless, our results differed largely from the concept of ADL predominantly relying on health-related factors and IADL being mainly based on psychosocial and economic factors [23, 32]. ADL and IADL coincided in many ways, instead of representing two complementing albeit distinct concepts. First, both ADL and IADL disability displayed an inverse correlation with the ‘Physical Health’ domain of QOL. While ADL/IADL disability led to lower scores in the domain ‘Physical Health’, ADL/IADL disability became less likely with higher scores in ‘Physical Health’. This is also in accordance with literature [47, 56]. Second, both ADL and IADL disability had an adverse association with the facet ‘Sensory Abilities’ and the domain ‘Psychological’. Considering the abovementioned concept, one would have expected a segregation with only ADL being linked to the former and solely IADL to the latter. Third, both ADL and IADL disability were significantly accompanied by a low functional status according to the TUG which primarily relies on physical functioning [125]. Forth, neither ADL nor IADL were significantly influenced by the nutritional status which is unanticipated given earlier reports and a supposed sequence of malnutrition leading to frailty which then leads to disability [133–135]. However, descriptive statistics reveal that among the 1375 included subjects only 13 were considered to be malnourished according to the MNA. This explains the insignificance of nutritional status in this sample. Fifth, both ADL and IADL disability were significantly related to the cognitive status and weak performances on the DemTect indicating possible dementia. Sixth, ‘Income’ affected neither ADL nor IADL. Perhaps this variable is solely decisive below a certain threshold which was not relevant in this sample [101]. Seventh, only ADL was associated with the variable ‘Social Participation’. In fact, ADL disability led to less complaints about lacking social participation. This might be surprising considering the conviction that low social participation is associated with disability [54, 82]. Applying the model of ‘Selective Optimization with Compensation’ helps explaining this phenomenon: While the level of social participation generally declines with increasing age, ADL disability might force the concerned elderlies to ‘select’ by setting new priorities more closely related to the disability, instead of focusing on the deficient social participation [136, 137]. However, the facet ‘Social Participation’ was indeed negatively associated with both ADL and IADL disability. This seems reasonable as both ADL and IADL independence as well as a sufficient nutritional and functional status are essential for involvement in social activities [138–140]. Moreover the facet ‘Social Participation’ was also related to an appropriate income which is the financial basis for some leisure activities (i.e., attending sport events, going on holidays) [141]. Actively engaging with others may help preventing disability and foster the QOL of the elderly [69, 142, 143].

Nonetheless, several results were in accordance with the idea of only IADL building mainly on psychosocial and cognitive resources. First, an intermediate education level exerted a significant protective effect only on IADL, but not on ADL [31]. Interestingly, having attained a high level of education remained insignificant. This could be a consequence of only 10.4% of the sample being assigned to the category ‘high education’, and smaller sample sizes making it harder to reach statistical significance [144]. Second, the domain ‘Social Relationships’ was solely significantly linked to IADL. Albeit in an unexpected direction as a higher QOL regarding ‘Social Relationships’ was associated with IADL disability. The same applied to the variable ‘Social Support’. Despite controversy over the influence of social support on ADL/IADL in the scientific literature, some previous studies revealed similar associations like those reported in this study [83, 145]. An explanation might be that having social support is an indicator for a prevalent or emerging disability [69, 146].

The domain ‘Physical Health’ overlapped mostly with ADL which was displayed in terms of its dependence on ADL disability and aligned associations with IADL disability, ‘Social Support’ and ‘Social Participation’, a reduced functional and nutritional status. However, in contrast to ADL, an appropriate income was associated with higher scores on ‘Physical Health’ which is in line with previous findings [62, 118]. Financial capability may help gaining an advantage within the public health system getting the best care available which then manifests itself in vigorous health and a higher (physical) QOL [80, 147].

Multivariate analysis revealed that both age and sex (partly) exert a significant influence on disability and physical QOL. First, an increased age was only significantly related to IADL disability, neither to ADL disability nor to the domain ‘Physical Health’. Especially the latter contradicts earlier findings [57, 80, 118]. In contrast to IADL, ADL and ‘Physical Health’ rather rely on the geriatric status which is predominantly founded on the biological age, not on the chronological age [60]. Second, women had lower odds of being IADL disabled (OR: 0.52). This was unanticipated, as similar results are rare and most studies report higher odds for women [35, 70, 148]. Interestingly, according to separate analysis of means (data not shown, an additional file shows this analysis in more detail (see Additional file 5)), there was no difference in terms of age between the two sexes (*p* value 0.5389). This would have been a plausible explanation considering the aforementioned outcome concerning age and IADL.

If IADL disability generally precedes ADL disability, and men are more likely to be IADL disabled, men will be expected to be more likely ADL disabled as well. However, this was not the case in this sample. In fact, women had higher odds of being ADL disabled (OR: 1.72), and scored lower on the domain ‘Physical Health’. Previous research has suggested that men face higher death rates among the studied age groups, while women experience higher incidence rates of ADL disability [42, 91, 149]. Higher death rates among men then lead to a selection of ‘the fittest’ men who in turn display lower ADL disability rates than their female counterparts [150]. Possible reasons are potentially different disability pathways due to physiological differences between both sexes (i.e., hormonal, childbirth) and a distinct structure of reality of life [151–153]. A female share of 64% in this study sample may support this theory of women outliving men at the expense of a higher prevalence of ADL disability [152]. Nevertheless, the selection of ‘the fittest’ men in this sample could also be due to disabled men relocating earlier to retirement homes than their female counterparts and therefore missing in this study according to the exclusion criteria. As a result of the cross-sectional nature of this study and unavailability of further relevant health-related information such as depression, pain and physical activity, it was not possible to clarify the following: First, whether IADL precedes ADL disability and if so, whether there is a direct mechanism leading from IADL to ADL disability [22, 34]. Second, whether the observed associations of age/sex with ADL/IADL are due to a direct influence, or whether age/sex function as mediator variables [32, 151].

This study has several limitations. One major limitation is its cross-sectional nature which does not allow assumptions about causality and generalisability to older populations [154] and which does not consider the time of presence of the determinants of disability and quality of life in the aging process. Another limitation concerns the healthy participation effect caused by the exclusion of 2741 subjects due to incomplete baseline data. Another shortcoming of this study concerns the dichotomisation of ADL and IADL ignoring different levels of disability [41, 45]. Moreover, interrater reliability regarding the examiners has to be discussed despite a training course prior to testing [155].

The study has various strengths as well. First, it is a multi-centre study in an actual care setting comprising eight certified practice networks including their GP practices. Despite the study’s cross-sectional entity, this fact enhances its representativity for older adults [156]. Second, it encompasses a large study population reducing the impact of accidental factors and raising the statistical power [144]. Third, the simultaneous exploration of the three concepts of ADL, IADL and QOL, and the study’s multifactorial examination of their determinants as well as the interactions among ADL, IADL and QOL. Forth, using both the WHOQOL-BREF and the WHOQOL-OLD to assess QOL in older people. Despite the WHOQOL-OLD’s proven psychometric properties there is still a scarcity of studies using this questionnaire [62, 157]. Hence, this study’s results may be particularly useful for the classification of future results in terms of the WHOQOL-OLD.

As the R^2^ values of our 14 multiple regression models ranged between 0.05 and 0.39, future research should focus on significant independent variables not included in this study. These comprise health care use and chronic medical conditions such as vascular disease, impairment of vision and hearing, pain, history of falls, polypharmacy and depression as well as their level of severity [40, 60]. Additionally, lifestyle-related factors like physical activity and consumption of alcohol and smoking should be assessed as well [54, 58].

This study detected and approved several potential determinants of disability and quality of life in the elderly. Attention should be drawn to prevention schemes as many of these determinants like the nutritional and functional status, social participation and education appear to be at least partly modifiable.

## Supplementary Information

**Additional file 1.** Bivariate Analysis of ADL.

**Additional file 2.** Bivariate Analysis of IADL.

**Additional file 3.** Multivariate Analysis – Effect Likelihood Ratio Tests (ADL, IADL, WHOQOL-BREF, WHOQOL-OLD).

**Additional file 4.** Bivariate Analysis of WHOQOL-BREF, WHOQOL-OLD.

**Additional file 5.** One-way Analysis of Age by Sex.

## Data Availability

The datasets used and/or analysed during the current study are available from the corresponding author on reasonable request.

## Abbreviations

ADL: activities of daily living
BI: Barthel Index
CCA: complete case analysis
DeGEval: German Evaluation Society
DFG: German Research Foundation
GEP: Good Epidemiological Practice
HRQOL: health-related quality of life
IADL: instrumental activities of daily living
ICF: International Classification of Functioning, Disability and Health
MNA: Mini Nutritional Assessment
OR: Odds Ratio
QOL: quality of life
RubiN: Regional ununterbrochen betreut im Netz
TUG: Timed Up-and-Go Test
WHO: World Health Organization
