# Supplementary material for: Multivariate analysis of independent determinants of ADL/IADL and quality of life in the elderly
Source: BMC Geriatr. 2022 Nov 23;22:894. doi: 10.1186/s12877-022-03621-3 (PMC9682836; doi:10.1186/s12877-022-03621-3)
Supplement: Supplementary file 5 — Additional file 5. One-way Analysis of Age by Sex. [file 12877_2022_3621_MOESM5_ESM.pdf]

Oneway Analysis of Age By Sex_Code

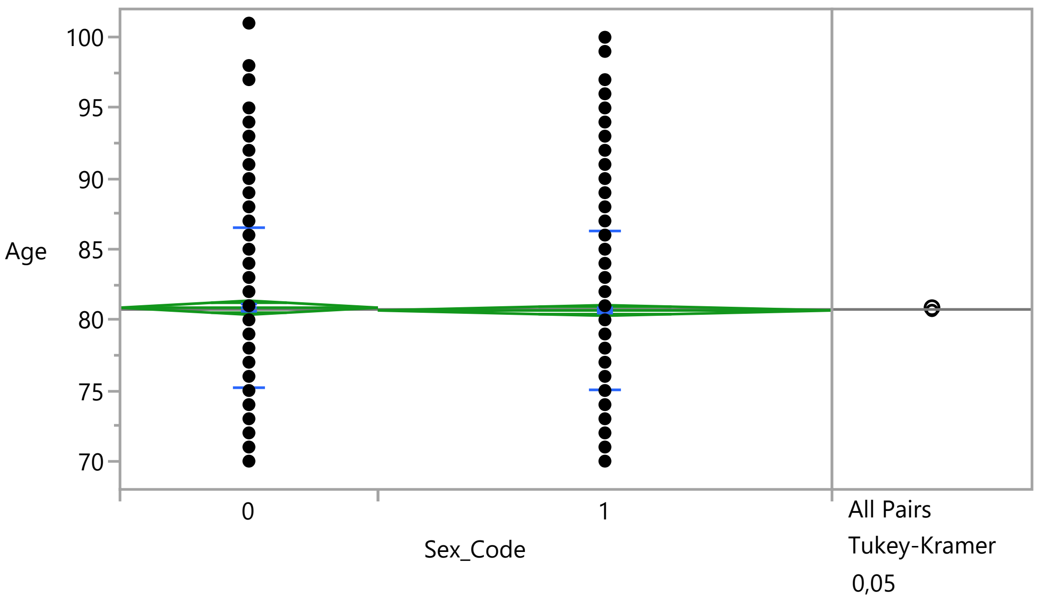

Oneway Anova

Summary of Fit

Rsquare	0,000275
Adj Rsquare	-0,00045
Root Mean Square Error	5,637006
Mean of Response	80,73745
Observations (or Sum Wgts)	1375

Pooled t Test

1-0

Assuming equal variances

Difference	-0,19440	t Ratio	-0,61462
Std Err Dif	0,31629	DF	1373
Upper CL Dif	0,42606	Prob > |t|	0,5389
Lower CL Dif	-0,81486	Prob > t	0,7305
Confidence	0,95	Prob < t	0,2695

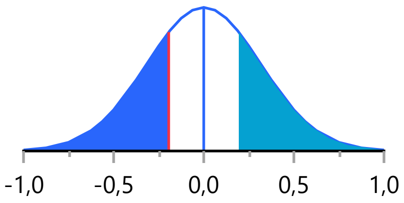

Analysis of Variance

Source	DF	Sum of Squares	Mean Square	F Ratio	Prob > F
Sex_Code	1	12,004	12,0037	0,3778	0,5389
Error	1373	43628,217	31,7758		
C. Total	1374	43640,221			

Means for Oneway Anova

Level	Number	Mean	Std Error	Lower 95%	Upper 95%
0	498	80,8614	0,25260	80,366	81,357
1	877	80,6670	0,19035	80,294	81,040

Std Error uses a pooled estimate of error variance

Oneway Analysis of Age By Sex_Code

Means and Std Deviations

Level	Number	Mean	Std Dev	Std Err Mean	Lower 95%	Upper 95%
0	498	80,861446	5,6631535	0,253772	80,362848	81,360044
1	877	80,667047	5,6221165	0,1898454	80,294442	81,039652

t Test

1-0

Assuming unequal variances

Difference	-0,19440	t Ratio	-0,61339
Std Err Dif	0,31693	DF	1026,537
Upper CL Dif	0,42750	Prob > |t|	0,5398
Lower CL Dif	-0,81629	Prob > t	0,7301
Confidence	0,95	Prob < t	0,2699

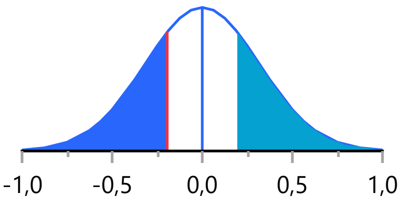

Means Comparisons

Comparisons for all pairs using Tukey-Kramer HSD

Confidence Quantile

q*	Alpha
1,96169	0,05

HSD Threshold Matrix

Abs(Dif)-HSD

	0	1
0	-0,70078	-0,42607
1	-0,42607	-0,52807

Positive values show pairs of means that are significantly different.
